# Burden of Adults Hospitalized With Group B Streptococcal Infection

**DOI:** 10.1093/infdis/jiaa110

**Published:** 2020-03-19

**Authors:** John M McLaughlin, Paula Peyrani, Stephen Furmanek, Farid L Khan, Angela Quinn, Luis Jodar, Julio Ramirez, David L Swerdlow

**Affiliations:** 1 Pfizer Vaccines, Collegeville, Pennsylvania, USA; 2 Division of Infectious Diseases, University of Louisville, Louisville, Kentucky, USA

**Keywords:** Group B *Streptococcus* (GBS), noninvasive, incidence, adults, epidemiology, risk factor

## Abstract

**Background:**

The burden of noninvasive group B *Streptococcus* (GBS) infections in adults is unknown. We determined population-based rates of hospitalization where invasive or noninvasive GBS infections were identified among US adults in a defined catchment area.

**Methods:**

We identified adults with clinical and laboratory-confirmed evidence of GBS infection from January 2014 through December 2016 from 6 hospitals in Louisville, Kentucky. Invasive disease was defined as GBS isolated from a normally sterile site.

**Results:**

Among 1076 adults with GBS infection, the median age was 52 years, 51% were male, and 89% had ≥1 chronic medical condition. The most prevalent infection sites were skin and soft tissue (39%), urinary tract (23%), bone and joint (16%), and bloodstream (11%). Forty percent of infections were polymicrobial. The annual incidence of GBS-associated hospitalization was 73 per 100 000 adults and 68 and 100 per 100 000 for patients aged 18–64 and ≥ 65 years, respectively. For every invasive GBS infection, 3.7 noninvasive infections occurred.

**Conclusions:**

Our population-based study outlines the full burden of GBS-associated hospitalization in adults and found incidence rates comparable to those of pneumococcal disease, where vaccines are recommended. Noninvasive disease was 3–4 times more common than invasive disease, suggesting that the GBS burden among adults is considerably greater than previously recognized.

## Teaser

Adult group B *Streptococcus* (GBS) burden is considerably greater than previously recognized, and there is a need to develop ways to preventing GBS among the growing population of adults who are older or have chronic medical conditions.

Group B *Streptococcus* (GBS) is a well-recognized cause of infection in neonates and pregnant women [1–3]. GBS also causes invasive infections in nonpregnant adults, especially among the elderly and adults with chronic medical conditions [4–8], resulting in significant morbidity and mortality rates [8–10]. Furthermore, contemporary reports from developed countries suggest that the incidence of invasive GBS disease in adults is increasing [5, 8, 11, 12].

Studies describing the burden of GBS in adults to date have primarily focused on invasive disease because existing surveillance systems rely on collection of blood cultures [8, 13]. However, GBS also causes many noninvasive infections of the skin and soft tissue, bone and joint, and urinary and respiratory tracts [11, 12]. Describing the incidence of these infections is more challenging because GBS may colonize skin and mucosal surfaces and may be isolated from infected sites along with other pathogens [14–17]. Thus, no studies to date have characterized the burden of noninvasive GBS infections in adults using a population-based approach. This type of study is needed to elucidate the full spectrum of the GBS disease burden in adults and inform future treatment and prevention strategies. We determined population-based rates of hospitalization where invasive or noninvasive GBS infections were identified among US adults living in a defined catchment area and extrapolated our results to the entire US population.

## METHODS

### Design, Setting, and Participants

We identified GBS infections among adults ≥18 years of age by retrospectively reviewing laboratory and medical records from 6 hospitals in Louisville, Kentucky, between 1 January 2014 and 31 December 2016. Data describing demographic and clinical characteristics (eg, chronic medical conditions) were collected for each patient. Institutional review board approval (no. 17.0542) was obtained by each participating center. The requirement for informed consent was waived.

Louisville is the largest city in Kentucky and sits on the Ohio River along the Indiana border. At the time of our study, Louisville’s population was generally similar to the United States in terms of demographics and the prevalence of underlying chronic medical conditions, including diabetes and obesity, based on data from the Behavioral Risk Factor Surveillance System (BRFSS). BRFSS is an annual survey conducted by the Centers for Disease Control and Prevention (CDC) that provides US-specific and Louisville Metropolitan Statistical Area–specific estimates [18]. 

Differences between the US population and Louisville included the facts that Louisville had a higher proportion of adults who were white (81% vs 71% for US population) and who currently smoked (22% vs 15%) and had fewer Hispanic adults (4% vs 19%) [18]. Louisville has 9 adult acute care hospitals, of which we included 6 for epidemiological description of GBS infections: KentuckyOne Health Jewish Hospital, the University of Louisville hospital, and the 4 adult hospitals in the Norton Healthcare system. For calculations of incidence, Jewish Hospital was excluded because not all patients with GBS were identified. The University of Louisville hospital identified all patients with GBS but for only 25 of 36 study months (1 April 2014 through 30 April 2016). The 5 hospitals included in incidence calculations accounted for 55% of all hospital admissions in the catchment area (Supplementary Table 1).

### Definition of GBS Infection

Patients had to have GBS isolated from culture obtained during hospitalization with (1) clinical or laboratory evidence of local signs and symptoms of infection or (2) systemic inflammatory response (Supplementary Table 2). Instances in which GBS was isolated from culture without local or systemic evidence of infection were categorized as colonization (only) and were excluded. Pregnant women meeting criteria for GBS infection were included (except for asymptomatic pregnant women with positive screening cultures). Infection sites were defined using clinical and laboratory criteria adapted from *Mandell, Douglas, and Bennett’s Principles and Practice of Infectious Diseases* [19] (Supplementary Table 2). 

If GBS was isolated from >1 clinical site, the most life-threatening or invasive site was considered the “most serious” infected site (eg, necrotizing fasciitis of the leg was considered the “most serious” site in a patient that also had cellulitis). To help establish these clear definitions of GBS infection, we consulted several experts (see Acknowledgments). Each case was reviewed by 2 infectious disease specialists (P. P. and J. R.). In instances where the type of GBS infection or the most serious site was not immediately clear, a third infectious disease expert was consulted (D. L. S.). For the most challenging cases, external experts who were not part of the study were also consulted (see Acknowledgments). Discrepancies between specialists were resolved through discussion until consensus was reached. Invasive disease was defined in accordance with CDC invasive bacterial surveillance criteria as GBS isolated from a normally sterile site, such as blood, bone, an internal body site (eg, specimen obtained from brain, lymph node, surgical site, or aspirate), or cerebrospinal, pleural, peritoneal, or pericardial fluid [20]. Infections such as osteomyelitis or necrotizing myositis were classified as noninvasive when the infection developed from another noninvasive contiguous site, such as an open wound. Polymicrobial infection was defined as the presence of GBS and ≥1 other organism in the same culture within the most serious site.

### Clinical Outcomes

For each GBS infection, we recorded the length of hospital stay and the proportion that were admitted to the intensive care unit (ICU), required mechanical ventilation, or died from any cause during hospitalization (in-hospital mortality rate) or within 30 days after hospital admission if discharged alive. Hospital-acquired infections were defined as those that occurred ≥48 hours after admission and did not seem to be incubating at the time of admission.

### Statistical Analysis

We compared invasive with noninvasive infections and monomicrobial with polymicrobial infections, using χ ^2^ tests. We estimated annual rates of GBS infection by dividing the number of GBS cases occurring among permanent residents of the catchment area (most serious site only) identified across the 5 hospitals used for calculating incidence by US Census population estimates for the catchment area. We adjusted the number of GBS infections for the estimated proportion of adult admissions in Louisville occurring in the 5 study hospitals (ie, market share). Age-specific market share data were obtained from the statewide Inpatient Records Database (Kentucky Cabinet for Health and Family Services). 

We also adjusted incidence estimates for the shortened period for which the University of Louisville Hospital recruited study participants (Supplementary Table 1). For calculation of risk group–specific incidence rates, the prevalence of chronic medical conditions in the catchment area were obtained from the Louisville Metropolitan Statistical Area-specific BRFSS [18]. Incidence rates were not age standardized to the US population, because the age distribution of adults in Louisville was nearly identical to that of the United States. Rates were presented for GBS infections both including and excluding noninvasive polymicrobial infections (where the role of GBS as the primary cause of infection may not be clear [14–17]). All analyses were performed with SAS software, version 9.4 (SAS Institute), or Stata software, version 14.0 (StataCorp).

## RESULTS

### Description of GBS Infections

We identified positive GBS cultures in 1428 hospitalized patients, of which 352 (25%) were deemed colonization (only). Of the remaining 1076 patients with GBS infection, 549 (51%) were male, 786 (73%) were white, 263 (24%) were black, and 961 (89%) had ≥1 chronic medical condition, with diabetes (633; 59%), obesity (585; 54%), coronary artery disease (221; 21%), and chronic renal disease (217; 20%) the most common. The median age was 52 years. Forty-five patients (4%) were pregnant or puerperal women. Only 38 (4%) were hospital acquired. The median hospital length of stay was 5 days. Twenty percent of patients (213 of 1076) were admitted to the ICU (Table 1). All-cause in-hospital deaths occurred in 30 of 1076 (3%). Six additional patients were discharged but died within 30 days of admission.

**Table 1.** Characteristics of Patients With Group B *Streptococcus* Infections^a^

Patient Characteristics	Patients, No. (%)			*P* Value
	All Patients (n = 1076)	Invasive Infection (n = 227)	Noninvasive Infection (n = 849)	
Demographics				
Year of study				
2014	293 (27)	60 (26)	233 (27)	.88
2015	388 (36)	85 (37)	303 (36)	
2016	395 (37)	82 (36)	313 (37)	
Age group, y				
18–49	452 (42)	68 (30)	384 (45)	<.001
50–64	388 (36)	101 (44)	287 (34)	
65–74	138 (13)	25 (11)	113 (13)	
≥75	98 (9)	33 (15)	65 (8)	
Sex				
Female	527 (49)	85 (37)	442 (52)	<.001
Male	549 (51)	142 (63)	407 (48)	
Race^b^				
White	786 (73)	175 (77)	611 (72)	.29
Black	263 (24)	47 (21)	216 (26)	
Other	24 (2)	4 (2)	20 (2)	
Ethnicity^c^				
Hispanic or Latino	26 (2)	3 (1)	23 (3)	.47
Not Hispanic or Latino	1043 (98)	223 (99)	820 (97)	
Body mass index^d^				
Underweight	40 (4)	6 (3)	34 (4)	.72
Normal weight	218 (20)	48 (21)	170 (20)	
Overweight	228 (21)	45 (20)	183 (22)	
Obesity	585 (54)	127 (56)	458 (54)	
Class 1	216 (20)	45 (20)	171 (20)	
Class 2	155 (14)	30 (13)	125 (15)	
Class 3	214 (20)	52 (23)	162 (19)	
Chronic medical conditions				
Any	961 (89)	209 (92)	752 (89)	.13
Diabetes mellitus	633 (59)	118 (52)	515 (61)	.02
Chronic renal disease	217 (20)	57 (25)	160 (19)	.04
Congestive heart failure	163 (15)	51 (22)	112 (13)	<.001
Coronary artery disease	221 (21)	51 (22)	170 (20)	.42
Peripheral vascular disease	147 (14)	32 (14)	115 (14)	.83
Stroke	101 (9)	17 (7)	84 (10)	.27
COPD	139 (13)	28 (12)	111 (13)	.77
Liver disease	74 (7)	29 (13)	45 (5)	<.001
Neoplastic disease	70 (7)	19 (8)	51 (6)	.20
HIV/AIDS	13 (1)	3 (1)	10 (1)	.74
Health behaviors				
Nursing home resident	66 (6)	20 (9)	46 (5)	.06
Current smoker	305 (28)	59 (26)	246 (29)	.37
Alcoholism	54 (5)	17 (7)	37 (4)	.06
Intravenous drug use	29 (3)	4 (2)	25 (3)	.49
Severity of infection				
ICU admission	213 (20)	69 (30)	144 (17)	<.001
Mechanical ventilation	129 (12)	42 (19)	87 (10)	<.001
Hospital acquired	38 (4)	10 (4)	28 (3)	.42
Pathogen results				
Monomicrobial	648 (60)	174 (77)	474 (56)	<.001
Polymicrobial	428 (40)	53 (23)	375 (44)	

Abbreviations: AIDS, acquired immunodeficiency syndrome; COPD, chronic obstructive pulmonary disease; HIV, human immunodeficiency virus; ICU, intensive care unit.

^a^If group B *Streptococcus* was isolated from >1 clinical site in the same patient, the most life-threatening or deepest site of infection was considered the most serious infected site.

^b^Race was missing for 3 patients.

^c^Ethnicity was missing for 7 patients.

^d^Underweight was defined as body mass index (BMI; calculated as weight in kilograms divided by height in meters squared) <18.5, normal weight as BMI 18.5–24.9, overweight as BMI 25.0–29.9, obesity as BMI ≥30.0, class 1 obesity as BMI 30.0–34.9, class 2 obesity as BMI 35.0–39.9, and class 3 obesity as BMI ≥40.0. BMI was missing for 8 patients.

Among the 1076 patients with GBS infection, GBS was isolated from 1784 samples. Most GBS isolations (983 of 1784 [55%]) were taken from skin and soft tissue, followed by urinary tract (269 of 1784 [15%]), bone and joint (248 of 1784 [14%]), and bloodstream (141 of 1784 [8%]) (Table 2).

**Table 2.** Anatomic Site for All Group B *Streptococcus* (GBS) Isolations (n = 1784) and for the Most Serious Sites of GBS Infection (n = 1076)^a^

Site of GBS Isolation	Isolations, No. (%)	
	All GBS Isolations (n = 1784)	Most Serious GBS Infections (n = 1076)
Skin and soft tissue	983 (55)	423 (39)
Urinary tract	269 (15)	252 (23)
Bone and joint	248 (14)	173 (16)
Bacteremia	141 (8)	115 (11)
Respiratory tract	70 (4)	57 (5)
Cardiovascular system	27 (2)	27 (3)
Intra-abdominal	24 (1)	17 (2)
Reproductive system	18 (1)	9 (1)
Central nervous system	4 (<1)	3 (<1)^b^

Abbreviation: GBS, group B *Streptococcus.*

^a^If GBS was isolated from >1 clinical site in the same patient, the most life-threatening or deepest site of infection was considered the most serious infected site.

^b^The instance in which a central nervous system isolate was not the most serious site of GBS infection was a patient with fluid surrounding the reservoir of a pain pump in the subcutaneous tissue (ie, seroma, resorbing hematoma, or abscess).

When analyses were restricted to the most serious site, skin and soft-tissue infections were still most common (423 of 1076 [39%]). Urinary tract (252 of 1076 [23%]), bone and joint (173 of 1076 [16%]), and bloodstream (115 of 1076 [11%]) infections comprised the remaining majority (Table 2). The 10 most common diagnoses were skin abscess, treated bacteriuria (abnormal urinalysis and antibiotic prescribed), osteomyelitis, secondary bacteremia, skin ulcer, necrotizing myositis, pyelonephritis, cystitis, necrotizing fasciitis, and community-acquired pneumonia, accounting for 84% (Table 3 and Supplementary Table 2). Results were similar when pregnant or puerperal women were excluded from the analyses (data not shown).

**Table 3.** Clinical Diagnoses for the Most Serious Group B *Streptococcus* Infections (n = 1076)^a^

Clinical Diagnosis	Most Serious Infections, No. (%)		
	All (n = 1076)	Invasive (n = 227)	Noninvasive (n = 849)
Abscess	228 (21)	2 (1)	226 (27)
Treated bacteriuria^b^	153 (14)	0 (0)	153 (18)
Osteomyelitis	134 (12)	6 (3)	128 (15)
Secondary bacteremia	95 (9)	95 (42)	0 (0)
Ulcer	71 (7)	0 (0)	71 (8)
Necrotizing myositis	55 (5)	17 (7)	38 (4)
Pyelonephritis	51 (5)	0 (0)	51 (6)
Cystitis	48 (4)	0 (0)	48 (6)
Necrotizing fasciitis	38 (4)	9 (4)	29 (3)
Community-acquired pneumonia	35 (3)	0 (0)	35 (4)
Cellulitis	32 (3)	0 (0)	32 (4)
Primary bacteremia	20 (2)	20 (9)	0 (0)
Septic arthritis	20 (2)	20 (9)	0 (0)
Endocarditis	19 (2)	19 (8)	0 (0)
Prosthetic joint infection	19 (2)	19 (8)	0 (0)
Hospital-acquired pneumonia	11 (1)	0 (0)	11 (1)
Tendinitis	7 (1)	1 (<1)	6 (1)
Acute exacerbation of chronic bronchitis	6 (1)	0 (0)	6 (1)
Line infection	6 (1)	6 (3)	0 (0)
Peritonitis	5 (<1)	2 (1)	3 (<1)
Chorioamnionitis	4 (<1)	0 (0)	4 (<1)
Spontaneous bacterial peritonitis	4 (<1)	4 (2)	0 (0)
Empyema	3 (<1)	3 (1)	0 (0)
Endometritis	3 (<1)	0 (0)	3 (<1)
Infected graft	2 (<1)	2 (1)	0 (0)
Endophthalmitis	1 (<1)	1 (<1)	0 (0)
Epididymitis	1 (<1)	0 (0)	1 (<1)
Epidural abscess	1 (<1)	1 (<1)	0 (0)
Mastoiditis	1 (<1)	0 (0)	1 (<1)
Meningitis	1 (<1)	0 (0)	1 (<1)^c^
Pyomyositis	1 (<1)	0 (0)	1 (<1)
Sinusitis	1 (<1)	0 (0)	1 (<1)

^a^If group B *Streptococcus* was isolated from >1 clinical site in the same patient, the most life-threatening or deepest site of infection was considered the “most serious” infected site.

^b^Bacteriuria with abnormal urinalysis results and antibiotic prescribed by the treating physician.

^c^Patient with fluid surrounding the reservoir of a pain pump in the subcutaneous tissue (ie, seroma, resorbing hematoma, or abscess).

### Invasive Versus Noninvasive Infections

Of the most serious infections, 21% (227 of 1076) were invasive. The most common invasive sites were bacteremia (115 of 227 [51%]), bone and joint (45 of 227 [20%]), skin and soft tissue (27 of 227 [12%]; eg, necrotizing fasciitis or myositis with samples collected during operative procedures), and the cardiovascular system (27 of 227 [12%]) (Supplementary Table 3). Invasive infections were more likely than noninvasive infections to occur in persons ≥75 years of age (33 of 227 invasive infections [15%] vs 65 of 849 noninvasive infections [8%]; *P *< .001), men (142 of 227 [63%] vs 407 of 849 [48%], respectively; *P *< .001), and those with congestive heart failure (51 of 227 [22%] vs 112 of 849 [13%]; *P *< .001), chronic renal disease (57 of 227 [25%] vs 160 of 849 [19%]; *P *= .04), or liver disease (29 of 227 [13%] vs 45 of 849 [5%]; *P *< .001) (Table 1). Invasive infections were more likely to require ICU admission (69 of 227 invasive infections [30%] vs 144 of 849 noninvasive infections [17%]; *P *< .001) and mechanical ventilation (42 of 227 [19%] vs 87 of 849 [10%], respectively; *P *< .001) and to be monomicrobial (174 of 227 [77%] vs 474 of 849 [56%]; *P *< .001) (Table 1). The most common diagnoses for invasive infections were secondary and primary bacteremia, septic arthritis, endocarditis, prosthetic joint infection, and necrotizing myositis and fasciitis, accounting for 88% of infections (Table 3 and Supplementary Table 2).

For noninvasive infections, skin and soft-tissue (396 of 849 [47%]), urinary tract (252 of 849 [30%]), bone and joint (128 of 849 [15%]), and respiratory tract (54 of 849 [6%]) infections were the most common (Supplementary Table 3). Noninvasive infections were more common than invasive infections in diabetics (515 of 849 [61%] vs 118 of 227 [52%], respectively; *P *= .02) (Table 1). The most common diagnoses for noninvasive infections were skin abscess, treated bacteriuria, osteomyelitis with open wound, skin ulcer, pyelonephritis, cystitis, necrotizing myositis with open wound, community-acquired pneumonia, accounting for 88% (Table 3).

On average, for every invasive GBS infection there were 3.7 noninvasive infections. This ratio was highest (5.6: 1) for adults 18–49 years of age and lowest (2.0: 1) for those ≥75 years of age.

### Polymicrobial Infection

Polymicrobial infection was identified in 428 of 1076 (40%) of the most serious sites, with 521 non-GBS isolates identified. The most common non-GBS organisms identified in polymicrobial infections were *Staphylococcus aureus* (204 of 521 [39%]), *Streptococcus* species (48 of 521 [9%]), and *Escherichia coli* (47 of 521 [9%]; Supplementary Table 4). Polymicrobial infections were more likely than monomicrobial infections to be skin and soft-tissue or bone and joint infections (331 of 428 polymicrobial infections [77%] vs 265 of 648 monomicrobial infections [41%]) and less likely to be bacteremia (18 of 428 [4%] vs 97 of 648 [15%], respectively) or urinary tract infections (42 of 428 [10%] vs 210 of 648 [32%]) (all *P *< .001; Supplementary Table 3). Polymicrobial infections were more likely than monomicrobial infections to occur in persons with noninvasive infections (375 of 428 [88%] vs 474 of 648 [73%], respectively; *P *< .001), in men (248 of 428 [58%] vs 301 of 648 [46%]; *P *< .001), and in those with diabetes (292 of 428 [68%] vs 341 of 648 [53%]; *P *< .001), peripheral vascular disease (79 of 428 [18%] vs 68 of 648 [10%]; *P* < .001), or stroke (51 of 428 [12%] vs 50 of 648 [8%]; *P *= .02), but were less common in those with liver disease (19 of 428 [4%] vs 55 of 648 [8%]; *P *= .01) (Supplementary Table 5). Among monomicrobial infections only, 2.7 noninvasive infections occurred for every invasive GBS infection.

### Incidence of GBS Infection

Among the 1076 hospitalizations in which GBS was identified, 684 were eligible for calculating incidence, corresponding to an annual rate of 73 (95% confidence interval [CI], 68–78) per 100 000 in all adults. Rates were 68 (95% CI, 63–74) and 100 (85–117) per 100 000 in adults 18–64 and ≥65 years of age, respectively. Rates for older adults were similar over time, but there was an annual increase in GBS rates among adults <65 years of age, from 52 (95% CI, 44–61) to 82 (72–94) per 100 000. The highest rate of GBS-associated hospitalization was seen among adults <65 years of age with diabetes, at 486 (95% CI, 437–540) per 100 000 annually (Table 4).

**Table 4.** Cases, Annual Incidence Rates, and Rate Ratios of the Most Serious Group B *Streptococcus* Infections by Patient Characteristics^a^

Patient Characteristics	Patients Aged 18–64 y			Patients Aged ≥65 y			Patients Aged ≥18 y		
	Cases	Annual Rate per 100 000	Rate Ratio (95% CI)	Cases	Annual Rate per 100 000	Rate Ratio (95% CI)	Cases	Annual Rate per 100 000	Rate Ratio (95% CI)
All patients	542	68	…	142	100	…	684	73	…
Demographics									
Year of study									
2014	136	52	1.0	49	109	1.0	185	60	1.0
2015	203	72	1.4 (1.1–1.7)	45	95	.9 (.6–1.3)	248	82	1.4 (1.1–1.6)
2016	203	82	1.6 (1.3–2.0)	48	104	.9 (.6–1.4)	251	78	1.3 (1.1–1.6)
Sex									
Female	259	64	1.0	74	91	1.0	333	69	1.0
Male	283	72	1.1 (.9–1.3)	68	112	1.2 (.8–1.6)	351	77	1.1 (1.0–1.3)
Race^b^									
White	346	54	1.0	112	89	1.0	458	60	1.0
Black	178	152	2.8 (2.3–3.4)	27	199	2.2 (1.5–3.4)	205	157	2.6 (2.2–3.1)
Other	18	40	.7 (.5–1.2)	2	…	…	20	41	.7 (.4–1,1)
Ethnicity^c^									
Not Hispanic or Latino	525	69	1.0	139	98	1.0	664	74	1.0
Hispanic or Latino	16	41	.6 (.4–1.0)	1	…	…	17	43	.6 (.4–.9)
Body mass index^d^									
Underweight	16	132	3.4 (1.9–6.0)	7	…	...	23	170	3.8 (2.4–6.1)
Normal weight	115	39	1.0	31	102	1.0	146	45	1.0
Overweight	117	45	1.1 (.9–1.5)	35	77	.8 (.5–1.2)	153	50	1.1 (.9–1.4)
Obesity	330	117	3.0 (2.4–3.8)	72	232	2.3 (1.5–3.5)	402	129	2.9 (2.4–3.5)
Class 1	119	77	2.0 (1.5–2.6)	33	161	1.6 (1.0–2.6)	152	87	1.9 (1.5–2.5)
Class 2	83	111	2.8 (2.1–3.8)	20	308	3.0 (1.7–5.3)	103	126	2.8 (2.2–3.7)
Class 3	128	247	6.3 (4.9–8.2)	19	476	4.6 (2.6–8.3)	147	263	5.9 (4.6–7.4)
Chronic medical conditions									
Diabetes mellitus									
No	216	30	1.0	66	60	1.0	282	34	1.0
Yes	326	486	16.1 (13.5–19.1)	76	240	4.0 (2.9–5.6)	402	409	12.0 (10.3–14.0)
Chronic renal disease									
No	441	57	1.0	95	72	1.0	536	59	1.0
Yes	100	401	7.0 (5.6–8.7)	47	473	6.6 (4.7–9.4)	147	421	7.1 (5.9–8.5)
Coronary artery disease									
No	460	60	1.0	81	70	1.0	541	62	1.0
Yes	82	279	4.6 (3.7–5.8)	61	236	3.4 (2.4–4.7)	143	259	4.2 (3.5–5.1)
Stroke									
No	503	65	1.0	112	89	1.0	615	68	1.0
Yes	39	165	2.5 (1.8–3.5)	30	181	2.0 (1.4–3.0)	69	171	2.5 (1.9–3.2)
COPD									
No	491	67	1.0	112	94	1.0	603	70	1.0
Yes	51	86	1.3 (1.0–1.7)	30	129	1.4 (.9–2.1)	81	98	1.4 (1.1–1.8)
Neoplasm									
No	515	67	1.0	132	114	1.0	647	73	1.0
Yes	27	86	1.3 (.9–1.9)	10	39	.3 (.2–.7)	37	66	.9 (.6–1.3)
Health behaviors									
Current smoker									
No	353	58	1.0	127	100	1.0	480	65	1.0
Yes	189	98	1.7 (1.4–2.0)	15	100	1.0 (.6–1.7)	204	98	1.5 (1.3–1.8)
Alcoholism									
No	511	69	1.0	136	99	1.0	647	74	1.0
Yes	31	56	.8 (.6–1.2)	6	…	…	37	60	.8 (.6–1.1)

Abbreviations: CI, confidence interval; COPD, chronic obstructive pulmonary disease.

^a^If group B *Streptococcus* (GBS) was isolated from >1 clinical site in the same patient, the most life-threatening or deepest site of infection was considered the most serious infected site. Rates were not calculated if the number of GBS cases was <10.

^b^Race was missing for 3 patients.

^c^Ethnicity was missing for 7 patients.

^d^Underweight was defined as body mass index (BMI; calculated as weight in kilograms divided by height in meters squared) <18.5, normal weight as BMI 18.5–24.9, overweight as BMI 25.0–29.9, obesity as BMI ≥30.0, class 1 obesity as BMI 30.0–34.9, class 2 obesity as BMI 35.0–39.9, and class 3 obesity as BMI ≥40.0; BMI was missing for 8 patients.

Black adults were 2.6 (95% CI, 2.2–3.1) times more likely than whites to acquire GBS, with a rate of 157 per 100 000 (Table 4). Annual rates among adults with chronic medical conditions were also higher. Rates for all GBS-associated hospitalizations and after exclusion of noninvasive, polymicrobial infections, respectively, were as follows, all per 100 000: chronic renal disease, 421 and 276; diabetes mellitus, 409 and 241; coronary artery disease, 259 and 158; history of stroke, 171 and 90; obesity, 129 and 83; chronic obstructive pulmonary disorder, 98 and 65; and current smoker status, 98 and 64. 

Rates increased linearly with increasing levels of obesity, with rates for all GBS-associated hospitalizations and after exclusion of noninvasive, polymicrobial infections of 87 and 57 per 100 000, respectively, for class 1 obesity, 126 and 82 per 100 000 for class 2 obesity, and 263 and 165 per 100 000 for class 3 obesity (Tables 4 and 5 and Supplementary Table 6). Overall, the rate of GBS excluding noninvasive, polymicrobial infections was 48 (95% CI, 43–52) per 100 000 among all adults and 43 (39–48) and 72 (59–87) per 100 000 among adults 18–64 and ≥65 years of age, respectively (Table 5 and Supplementary Table 6). The rate of invasive GBS was 15 (95% CI, 13–17) per 100 000 per year, with rates of 13 (11–16) and 25 (18–35) per 100 000 in adults aged 18–64 and ≥65 years, respectively (Supplementary Table 6).

**Table 5.** Annual Incidence of Group B *Streptococcus* (GBS) Infection in Select Populations, Including and Excluding Noninvasive Polymicrobial GBS Infections by Age Group^a^

Characteristic	All Most Serious GBS Infections (Excluding Noninvasive Polymicrobial Infections), No. per 100 000		
	Patients Aged 18–64 y	Patients Aged ≥65 y	Patients Aged ≥18 y
All	68 (43)	100 (72)	73 (48)
Black adults^a^	152 (92)	199 (119)	157 (95)
White adults^a^	54 (35)	89 (67)	60 (40)
Chronic renal disease	401 (238)	473 (375)	421 (276)
Diabetes	486 (280)	240 (156)	409 (241)
Coronary artery disease	279 (149)	236 (168)	259 (158)
Stroke	165 (74)	181 (114)	171 (90)
Obesity^b^	117 (74)	232 (171)	129 (83)
Class 1	77 (48)	161 (127)	87 (57)
Class 2	111 (71)	308 (216)	126 (82)
Class 3	247 (153)	476 (325)	263 (165)
COPD	86 (53)	129 (98)	98 (65)
Current smokers	98 (63)	100 (81)	98 (64)

Abbreviations: COPD, chronic obstructive pulmonary disease; GBS, group B *Streptococcus*.

^a^If GBS was isolated from >1 clinical site in the same patient, the most life-threatening or deepest site of infection was considered the most serious infected site. Rates were presented for all GBS infections and, in parentheses, excluding noninvasive polymicrobial infections (where the role of GBS as the primary cause of infection may not be clear).

^b^Race was missing for 3 patients.

^c^Obesity was defined as body mass index (BMI; calculated as weight in kilograms divided by height in meters squared) ≥30.0, class 1 obesity as BMI 30.0–34.9, class 2 obesity as BMI 35.0–39.9, and class 3 obesity as BMI ≥40.0. BMI was missing for 8 patients.

## DISCUSSION

To our knowledge, this is the first population-based study to determine the total burden of hospitalization where both invasive and noninvasive GBS infection was identified among adults. Previous studies have described the adult burden of invasive GBS, but hospitalization with noninvasive GBS disease was 3–4 times more common in our study. This finding suggests that the adult burden of GBS is considerably greater than previously recognized.

The annual incidence of hospitalization where GBS was identified from any source was 73 per 100 000 for all adults and reached 100 per 100 000 in adults ≥65 years of age. The in-hospital mortality rate was 3%. Applying these rates of infection and case fatality to the 2020 US adult population corresponds to an estimated 188 570 (95% CI, 175 290–202 710) GBS-related hospitalizations and 5660 (5260–6080) deaths annually. If noninvasive, polymicrobial infections (where the role of GBS as the primary cause of infection may not be clear [14–17]) were excluded, rates were 48 (95% CI, 43–52) and 72 (59–87) per 100 000 among adults ≥18 years and ≥65 years of age, respectively. These rates still translate to an estimated 122 960 (95% CI, 112 260–134 420) GBS-related hospitalizations and 3690 (95% CI, 3370–4030) deaths each year in the United States.

Rates for older adults were similar over the 3-year study period, but there was an increase in GBS rates among adults <65 years of age during the study. Consistent with previous reports [4–6], most GBS infections (89%) occurred in adults with chronic medical conditions, most commonly diabetes, obesity, coronary artery disease, and chronic renal disease. Compared with the general population, rates of GBS were 2–6 times higher in these patients, with incidence rates of GBS infection between 129 and 421 per 100 000. The highest rate of GBS-associated hospitalization was seen among adults <65 years of age with diabetes, reaching almost 500 per 100 000 annually. Patients in this age group were 16 times more likely to be hospitalized with GBS than those without diabetes. Although these conditions have previously been identified as risk factors for invasive GBS [4–6], our population-based study allowed us to evaluate rates of both invasive and noninvasive disease and to construct detailed age-specific and risk group–specific rates of GBS infection. Future studies should evaluate the interaction between potentially related chronic medical conditions (eg, diabetes, obesity, and renal disease) and rates of GBS infection.

Rates of GBS-related hospitalization in older adults and in adults with chronic medical conditions were comparable to or even higher than rates of pneumococcal disease for which vaccines are recommended. For example, the rate of hospitalization for invasive and noninvasive vaccine-type pneumococcal infection in adults ≥65 years of age was estimated to be 144 per 100 000 [21] in 2014 when 13-valent pneumococcal conjugate vaccine was universally recommended in this age group [22].

The overall annual rate of invasive GBS was 15 per 100 000 and is consistent with, albeit slightly higher than, previous estimates from other population-based studies of adults using similar laboratory-based surveillance methods, which ranged from 3 to 11 per 100 000 [4, 5, 8, 11, 18]. Our rate of invasive GBS infection among adults ≥65 years of age was 25 per 100 000, which is identical to estimates from the CDC Active Bacterial Core Surveillance program in the same age group and during the same time period [18]. In addition, our risk group–specific rates of invasive GBS among patients with diabetes and obesity were similar to rates in a recently published CDC report [6], further confirming the robustness of our estimates.

Polymicrobial infection was common, occurring in 40% of GBS infections. Most (77%) polymicrobial infections were skin and soft-tissue or bone and joint infections. *S. aureus*, which is also frequently associated with skin infection, was the most commonly identified pathogen in polymicrobial infections, occurring in 39%. The exact role of GBS in the pathogenesis of polymicrobial infections is not clear [14–17]. For this reason, we presented our results both including and excluding polymicrobial noninvasive infections, and we are planning future research to better characterize the nature of polymicrobial GBS infections.

Our study has limitations. Our study population was limited to a single US city and may not be generalizable. The adult population in Louisville, however, is generally similar to the US adult population in terms of demographics and the prevalence of underlying chronic medical conditions, including diabetes and obesity, with few exceptions (eg, higher smoking prevalence and fewer Hispanics) [18]. Although the network of Louisville hospitals included in our study have produced reliable estimates of pneumonia surveillance in the past [23], we included only 5 of 9 hospitals in the catchment area. However, we adjusted for market share to account for this limitation. Thus, our estimates should be unbiased if patients with GBS infection were not disproportionately referred to a particular hospital or group of hospitals in Louisville, which seems unlikely.

Our rates of GBS may be underestimated, given that GBS cases were identified via routine culture, and many patients may have been treated without cultures being performed. In addition, given the retrospective nature of our study, long-term follow-up was not conducted to assess rates of relapse or recurrence of GBS infection. Furthermore, there is an additional outpatient burden of noninvasive GBS disease that was not measured in our study and warrants future research.

It is unknown whether GBS infection was the primary reason for hospitalization in all cases with community-onset, which accounted for 96% of GBS infections. This distinction, however, is rarely made in studies of infectious disease incidence (eg, studies to define the burden and etiology of adult pneumonia) [24]. Nevertheless, we admit that determining the burden of noninvasive GBS is challenging, because distinguishing between GBS colonization and infection can be difficult, and because GBS may be one of several pathogens isolated from a given culture (ie, polymicrobial infections occur frequently). 

These complicating factors are key reasons the noninvasive burden of GBS in adults has never been described. To address these concerns and establish clear definitions of GBS infection, we consulted numerous experts. In addition, we collected exhaustive data describing clinical and laboratory findings for each patient, and each case was reviewed by multiple infectious disease specialists. Moreover, rates of GBS infections were presented both including and excluding noninvasive polymicrobial infections to ensure a range of plausible GBS incidence estimates was available. A final limitation is that GBS isolates were not serotyped, and future research is needed to determine the proportion of GBS disease that could be vaccine preventable. However, multivalent polysaccharide conjugate vaccines currently in development broadly target the most prevalent GBS serotypes [25].

Our population-based study outlines the full burden of hospitalization in adults in whom GBS infection was identified and found incidence rates comparable to those of pneumococcal disease where vaccines are recommended. Although previous studies have highlighted the growing burden of invasive GBS in adults, our study suggests for every invasive GBS infection requiring hospitalization, at least 3–4 hospitalized noninvasive infections also occur. Our results emphasize the importance of developing approaches for preventing GBS, especially among the growing population of adults who are older or have chronic medical conditions.

## Supplementary Data

Supplementary materials are available at The Journal of Infectious Diseases online. Consisting of data provided by the authors to benefit the reader, the posted materials are not copyedited and are the sole responsibility of the authors, so questions or comments should be addressed to the corresponding author.

jiaa110_suppl_Supplementary_Material

## Notes

***Acknowledgments.*** The authors thank Ruth Lynfield, MD, Kathy Como-Sabetti, MPH, and Brenda Jewell from the Minnesota Department of Health for their insight into how to characterize invasive group B *Streptococcus* infections; Kevin Yi, PhD, from Pfizer for his quality control review of the study data; David Rimland, MD, for reviewing the protocol and his insight into how to characterize invasive group B *Streptococcus* infections.

***Financial support.*** This work was supported by Pfizer.

***Potential conflicts of interest.*** J. M. M., P. P., F. L. K., A. Q., L. J., and D. L. S. are employees and shareholders of Pfizer, and S. F. and J. R. received funding from Pfizer for the conduct of the study. The sponsor was involved with the study concept and design, conduct, analysis, and interpretation of the data; drafting of the manuscript; and the decision to submit the manuscript for publication. All authors have submitted the ICMJE Form for Disclosure of Potential Conflicts of Interest. Conflicts that the editors consider relevant to the content of the manuscript have been disclosed.
