# Supplementary material for: Burden of Adults Hospitalized With Group B Streptococcal Infection
Source: J Infect Dis. 2020 Mar 19;224(7):1170–8. doi: 10.1093/infdis/jiaa110 (PMC8561246; doi:10.1093/infdis/jiaa110)
Supplement: jiaa110_suppl_Supplementary_Material [file jiaa110_suppl_Supplementary_Material.docx]

**SUPPLEMENTARY MATERIAL**

**Burden of Adults Hospitalized with Group B Streptococcal Infection**

**Table of Contents**

| Table S1 | Description of study hospitals | 3 |
| --- | --- | --- |
| Table S2 | Definition of GBS infection | 4 |
| Table S3 | Most-serious infection site by type of GBS infection (n=1076) | 6 |
| Table S4 | Non-GBS pathogens identified in most-serious site polymicrobial infections (n=521) | 7 |
| Table S5 | Comparison of monomicrobial and polymicrobial most-serious site of GBS infection (n= 1076) | 8 |
| Table S6 | Annual incidence rates of most-serious GBS infections by patient characteristic and type of infection | 10 |

**Supplemental Table 1.** Description of study hospitals

| Hospital characteristic | Jewish* | Norton Audubon | Norton Brownsboro | Norton Downtown | Norton Suburban | University of Louisville |
| --- | --- | --- | --- | --- | --- | --- |
| Study start | Jan 2014 | Jan 2014 | Jan 2014 | Jan 2014 | Jan 2014 | Apr 2014 |
| Study end | Dec 2016 | Dec 2016 | Dec 2016 | Dec 2016 | Dec 2016 | Apr 2016 |
| No. months participated | 36 | 36 | 36 | 36 | 36 | 25 |
| Observed cases |  |  |  |  |  |  |
| All cases | 103 | 307 | 136 | 222 | 156 | 152 |
| Louisville residents only | 64 | 251 | 79 | 144 | 113 | 97 |
| Adjusted incident cases | n/a | 251 | 79 | 144 | 113 | 140^†^ |
| Hospital market share^‡^ |  |  |  |  |  |  |
| 18 to 64 years | n/a | 12.3% | 6.0% | 16.7% | 15.6% | 8.9% |
| ≥65 years | n/a | 19.4% | 6.9% | 6.8% | 8.7% | 2.4% |

n/a= not applicable.

*Jewish hospital was not included in calculations of incidence because not all GBS patients were identified.

^†^Inflated by a factor of 36/25 to account for the months where cases were not accrued (i.e., University of Louisville study participation was 25 of 36 months from April 1, 2014 through April 30, 2016).
^‡^Age-specific market share of the study hospitals was estimated using data from the statewide Inpatient Records Database which is maintained by the Kentucky Cabinet for Health and Family Services.

**Supplemental Table 2.** Definition of GBS infection

| Isolation of GBS from culture  PLUS ≥1 Clinical or Laboratory Sign or Symptom of Local Infection  OR  ≥1 Systemic Inflammatory Response | |
| --- | --- |
| Clinical or Laboratory Sign or Symptom of Local Infection | Systemic Inflammatory Response |
| *Skin and soft tissue infection*   - Cellulitis: redness/warmth, tenderness, induration, purulent drainage - Necrotizing fasciitis, myositis, tendinitis, pyomyositis: redness/warmth, tenderness, induration, positive imaging studies - Ulcer: redness/warmth, tenderness, induration, purulent drainage in an area with an open wound - Abscess: redness/warmth, tenderness, induration, with or without purulent drainage with documentation in the medical record of the presence of an abscess   *Urinary tract infection*   - Cystitis: dysuria, suprapubic pain, pyuria - Pyelonephritis: back pain, hematuria, pyuria - Treated bacteriuria: bacteriuria with abnormal urinalysis and antibiotic prescribed by treating physician   *Bone and joint infection*   - Septic arthritis: redness, swelling, and/or tenderness over a joint area, positive imaging studies, abnormal synovia fluid study - Prosthetic join infection: presence of sinus tract with active drainage, swelling, and/or tenderness over a joint area, positive imaging studies - Osteomyelitis: presence of sinus tract with active drainage, swelling, and/or tenderness over a joint area, positive imaging studies   *Respiratory tract infection*   - Pharyngitis: sore throat - Sinusitis: facial pain, positive imaging studies - Otitis: otalgia - Acute exacerbation of chronic obstructive pulmonary disease (COPD): new or worsening cough, new or worsening respiratory secretions, shortness of breath, hypoxemia (oxygen saturation <90% on room) - Pneumonia: new or worsening cough, new or worsening respiratory secretions, shortness of breath, pleuritic chest pain, hypoxemia (oxygen saturation <90% on room air or PaFiO2 <250), positive imaging studies - Empyema with pleural effusion: positive imaging studies   *Intra-abdominal*   - Abscess: fevers, chills, abdominal tenderness, malaise - Spontaneous bacterial peritonitis: fevers, chills, nausea, vomiting, abdominal tenderness, malaise, abdominal pain, worsening ascites without evidence of another abdominal infected site - Secondary peritonitis: fevers, chills, nausea, vomiting, abdominal tenderness, malaise, abdominal pain with evidence of an infected contiguous site   *Central nervous system*   - Meningitis: headache, photophobia, neck stiffness, nausea, vomiting, abnormal CSF study - Brain abscess: headache, nausea, vomiting, positive imaging studies - Endophthalmitis: ocular pain, visual changes, positive imaging studies   *Cardiovascular*   - Endocarditis: positive blood cultures, positive imaging studies - Line infection: positive blood cultures, positive catheter tip culture - Infected graft: positive imaging studies   *Reproductive*   - Epididymitis/orchitis: redness/warmth, tenderness, induration, positive imaging studies - Chorioamnionitis: fevers, chills, abdominal pain in a pregnant woman - Endometritis: fevers, chills, abdominal pain - Pelvic abscess: fevers, chills, abdominal pain, positive imaging studies   *Bacteremia*   - Primary bacteremia: positive blood cultures without an identified source of infection - Secondary bacteremia: positive blood cultures with a source of infection | *Temperature changes*   - Fever - Hypothermia   *Cardiovascular changes*   - Tachycardia - Hypotension   *Respiratory Changes*   - Elevated respiratory rate   *White blood cell count changes*   - Leukocytosis - Leukopenia - Left shift   *Elevated inflammatory markers*   - Erythrocyte sedimentation rate - C-reactive protein - Procalcitonin |

**Supplemental Table 3.** Most-serious infection site by type of GBS infection (n=1076)

| Site of most-serious infection | Invasive (n= 227) | Non-invasive (n= 849) | Monomicrobial (n= 648) | Polymicrobial (n= 428) |
| --- | --- | --- | --- | --- |
|  | *n (%)* | | | |
| Skin and soft tissue | 27 (12) | 396 (47) | 183 (28) | 240 (56) |
| Urinary tract | 0 (0) | 252 (30) | 210 (32) | 42 (10) |
| Bone and joint | 45 (20) | 128 (15) | 82 (13) | 91 (21) |
| Bacteremia | 115 (51) | 0 (0) | 97 (15) | 18 (4) |
| Respiratory tract | 3 (1) | 54 (6) | 37 (6) | 20 (5) |
| Cardiovascular system | 27 (12) | 0 (0) | 24 (4) | 3 (1) |
| Intra-abdominal | 7 (3) | 10 (1) | 5 (1) | 12 (3) |
| Reproductive system | 1 (<1) | 8 (1) | 7 (1) | 2 (<1) |
| Central nervous system | 2 (1) | 1 (<1)* | 3 (<1) | 0 (0) |

GBS= Group B *Streptococcus.* If GBS was isolated from more than one clinical site, the most life-threatening or deepest site of infection was considered the ‘most-serious’ infected site.

*Patient with fluid surrounding the reservoir of a pain pump in the subcutaneous tissue (i.e., was seroma, resorbing hematoma, or abscess).

**Supplemental Table 4** Non-GBS pathogens identified in most-serious site polymicrobial infections (n=521)

| Pathogen by type | n (%) |
| --- | --- |
| Gram Positives | 325 (62) |
| *Staphylococcus aureus* | 204 (39) |
| MSSA | 109 (21) |
| MRSA | 91 (17) |
| Resistance unknown | 4 (<1) |
| *Streptococcus* | 48 (9) |
| *Enterococcus* | 28 (5) |
| Other *Staphylococcus* | 28 (5) |
| Other Gram positive | 17 (3) |
| Gram Negatives | 150 (29) |
| *Escherichia coli* | 47 (9) |
| Proteus | 22 (4) |
| *Pseudomonas* | 19 (4) |
| *Enterobacter* | 19 (4) |
| *Klebsiella* | 14 (3) |
| Other Gram negative | 29 (6) |
| Anaerobes | 28 (5) |
| Yeast | 12 (2) |
| Unknown | 6 (1) |

GBS= Group B *Streptococcus; MRSA=* methicillin-resistant *Staphylococcus aureus; MSSA=* methicillin-susceptible *Staphylococcus aureus.* If GBS was isolated from more than one clinical site, the most life-threatening or deepest site of infection was considered the ‘most-serious’ infected site.

**Supplemental Table 5.** Comparison of monomicrobial and polymicrobial most-serious site of GBS infection (n= 1076)

| Patient Characteristics | Monomicrobial (n= 648) | Polymicrobial (n= 428) |  | *P* value |
| --- | --- | --- | --- | --- |
| *Demographics* | *n (%)* | |  |  |
| Year of study |  |  |  | .50 |
| 2014 | 173 (27) | 120 (28) |  |  |
| 2015 | 228 (35) | 160 (37) |  |  |
| 2016 | 247 (38) | 148 (35) |  |  |
| Age |  |  |  | .16 |
| 18 to 49 | 272 (42) | 180 (42) |  |  |
| 50 to 64 | 222 (34) | 166 (39) |  |  |
| 65 to 74 | 86 (13) | 52 (12) |  |  |
| ≥75 | 68 (10) | 30 (7) |  |  |
| Sex |  |  |  | <.001 |
| Female | 347 (54) | 180 (42) |  |  |
| Male | 301 (46) | 248 (58) |  |  |
| Race* |  |  |  | .10 |
| White | 485 (75) | 301 (71) |  |  |
| Black | 145 (22) | 118 (28) |  |  |
| Other | 17 (3) | 7 (2) |  |  |
| Ethnicity^†^ |  |  |  | .65 |
| Hispanic or Latino | 18 (3) | 8 (2) |  |  |
| Not Hispanic or Latino | 626 (97) | 417 (98) |  |  |
| *Chronic Medical Conditions* |  |  |  |  |
| Any | 576 (89) | 385 (90) |  | .58 |
| Diabetes mellitus | 341 (53) | 292 (68) |  | <.001 |
| Obesity^‡^ | 353 (54) | 232 (54) |  | .87 |
| Chronic renal disease | 124 (19) | 93 (22) |  | .29 |
| Congestive heart failure | 97 (15) | 66 (15) |  | .83 |
| Coronary artery disease | 121 (19) | 100 (23) |  | .06 |
| Peripheral vascular disease | 68 (10) | 79 (18) |  | <.001 |
| Stroke | 50 (8) | 51 (12) |  | .02 |
| COPD | 90 (14) | 49 (11) |  | . 24 |
| Liver disease | 55 (8) | 19 (4) |  | .01 |
| Neoplastic disease | 43 (7) | 27 (6) |  | .83 |
| HIV/AIDS | 11 (2) | 2 (<1) |  | .09 |
| *Health Behaviors* |  |  |  |  |
| Nursing home resident | 40 (6) | 26 (6) |  | .95 |
| Current smoker | 178 (28) | 127 (30) |  | .44 |
| Alcoholism | 38 (6) | 16 (4) |  | .12 |
| Intravenous drug use | 14 (2) | 15 (4) |  | .18 |
| *Severity of infection* |  |  |  |  |
| ICU admission | 137 (21) | 76 (18) |  | .17 |
| Mechanical ventilation | 82 (13) | 47 (11) |  | .42 |
| Hospital-acquired | 22 (3) | 16 (4) |  | .77 |
| Invasive | 174 (27) | 53 (12) |  | <.001 |

COPD= chronic obstructive pulmonary disease; GBS= Group B *Streptococcus*; HIV/AIDS= human immunodeficiency virus or acquired immunodeficiency syndrome; ICU= intensive care unit. If GBS was isolated from more than one clinical site, the most life-threatening or deepest site of infection was considered the ‘most-serious’ infected site.

*Race was missing for 3 patients.

^†^Ethnicity was missing for 7 patients.

^‡^Obesity was defined as body mass index ≥30; body mass index was missing for 8 patients.

**Supplemental Table 6.** Annual incidence rates of most-serious GBS infections by patient characteristic and type of infection

|  | 18 to 64 years of age | | | | ≥65 years of age | | | | ≥18 years of age | | | |
| --- | --- | --- | --- | --- | --- | --- | --- | --- | --- | --- | --- | --- |
|  | Invasive | | Non-invasive | | Invasive | | Non-invasive | | Invasive | | Non-invasive | |
| Patient characteristic | All | Monomicrobial only | All | Monomicrobial only | All | Monomicrobial only | All | Monomicrobial only | All | Monomicrobial only | All | Monomicrobial only |
| All | 13 | 10 | 55 | 30 | 25 | 20 | 74 | 46 | 15 | 12 | 58 | 33 |
| *Demographics* |  |  |  |  |  |  |  |  |  |  |  |  |
| Year of study |  |  |  |  |  |  |  |  |  |  |  |  |
| 2014 | 10 | 7 | 42 | 23 | 31 | 24 | 78 | 47 | 13 | 9 | 47 | 27 |
| 2015 | 12 | 10 | 59 | 32 | 28 | 21 | 68 | 42 | 15 | 13 | 66 | 36 |
| 2016 | 17 | 14 | 65 | 36 | 20 | 17 | 84 | 54 | 17 | 13 | 62 | 36 |
| Sex |  |  |  |  |  |  |  |  |  |  |  |  |
| Female | 9 | 7 | 55 | 34 | 16 | 12 | 74 | 51 | 10 | 8 | 58 | 37 |
| Male | 17 | 13 | 55 | 27 | 37 | 31 | 75 | 40 | 19 | 16 | 58 | 28 |
| Race* |  |  |  |  |  |  |  |  |  |  |  |  |
| White | 11 | 9 | 43 | 24 | 21 | 17 | 68 | 46 | 13 | 11 | 47 | 27 |
| Black | 24 | 17 | 128 | 68 | -- | -- | 138 | -- | 28 | 20 | 129 | 67 |
| Other | -- | -- | 33 | 24 | -- | -- | -- | -- | -- | -- | 34 | 24 |
| Ethnicity^†^ |  |  |  |  |  |  |  |  |  |  |  |  |
| Not Hispanic  or Latino | 14 | 11 | 56 | 31 | 24 | 19 | 73 | 45 | 15 | 12 | 59 | 33 |
| Hispanic or  Latino | -- | -- | 39 | -- | -- | -- | -- | -- | -- | -- | 40 | 25 |
| Body mass index^‡^ |  |  |  |  |  |  |  |  |  |  |  |  |
| Underweight | -- | -- | 123 | 91 | -- | -- | -- | -- | -- | -- | 167 | 109 |
| Normal Weight | 9 | 6 | 30 | 18 | -- | -- | 74 | 45 | 11 | 8 | 34 | 21 |
| Overweight | 10 | 6 | 35 | 16 | -- | -- | 60 | 36 | 11 | 7 | 39 | 19 |
| Obesity | 21 | 18 | 96 | 53 | 61 | 52 | 171 | 110 | 25 | 22 | 104 | 58 |
| Class 1 | 13 | 12 | 64 | 35 | -- | -- | 117 | 83 | 17 | 15 | 70 | 41 |
| Class 2 | 13 | 13 | 97 | 57 | -- | -- | 231 | -- | 18 | 17 | 108 | 64 |
| Class 3 | 54 | 44 | 193 | 99 | -- | -- | 351 | -- | 59 | 50 | 204 | 106 |
| *Chronic Medical*  *Conditions* |  |  |  |  |  |  |  |  |  |  |  |  |
| Diabetes  mellitus |  |  |  |  |  |  |  |  |  |  |  |  |
| No | 7 | 6 | 24 | 15 | 20 | 17 | 40 | 28 | 8 | 7 | 26 | 17 |
| Yes | 84 | 59 | 402 | 195 | 45 | 31 | 195 | 111 | 72 | 50 | 337 | 169 |
| Obesity^‡^ |  |  |  |  |  |  |  |  |  |  |  |  |
| No | 9 | 6 | 34 | 19 | 16 | 12 | 53 | 32 | 10 | 7 | 37 | 21 |
| Yes | 21 | 18 | 96 | 53 | 46 | 38 | 127 | 81 | 24 | 21 | 100 | 56 |
| Chronic renal  disease |  |  |  |  |  |  |  |  |  |  |  |  |
| No | 10 | 8 | 47 | 27 | 15 | 14 | 57 | 34 | 11 | 9 | 48 | 28 |
| Yes | 97 | 73 | 305 | 142 | 161 | 108 | 312 | 214 | 114 | 82 | 307 | 162 |
| Coronary  Artery Disease |  |  |  |  |  |  |  |  |  |  |  |  |
| No | 11 | 9 | 49 | 28 | 17 | 15 | 53 | 34 | 12 | 10 | 50 | 29 |
| Yes | 68 | 45 | 211 | 82 | 65 | 46 | 171 | 103 | 66 | 46 | 193 | 91 |
| Stroke |  |  |  |  |  |  |  |  |  |  |  |  |
| No | 12 | 10 | 53 | 30 | 23 | 21 | 66 | 43 | 14 | 12 | 55 | 32 |
| Yes | 31 | 13 | 134 | 43 | -- | -- | 140 | 73 | 35 | 15 | 136 | 55 |
| COPD |  |  |  |  |  |  |  |  |  |  |  |  |
| No | 12 | 10 | 55 | 30 | 25 | 20 | 69 | 42 | 14 | 11 | 56 | 32 |
| Yes | 22 | 20 | 64 | 31 | -- | -- | 102 | 71 | 23 | 21 | 75 | 42 |
| Neoplasm |  |  |  |  |  |  |  |  |  |  |  |  |
| No | 12 | 9 | 55 | 30 | 29 | 24 | 84 | 52 | 14 | 11 | 59 | 33 |
| Yes | 31 | 31 | 55 | 33 | -- | -- | -- | -- | 21 | 19 | 45 | 29 |
| *Health Behaviors* |  |  |  |  |  |  |  |  |  |  |  |  |
| Current  smoker |  |  |  |  |  |  |  |  |  |  |  |  |
| No | 11 | 9 | 47 | 26 | 25 | 20 | 75 | 46 | 13 | 11 | 52 | 29 |
| Yes | 19 | 15 | 79 | 44 | -- | -- | 72 | -- | 20 | 15 | 79 | 44 |
| Alcoholism |  |  |  |  |  |  |  |  |  |  |  |  |
| No | 12 | 9 | 57 | 31 | 24 | 19 | 75 | 47 | 14 | 11 | 60 | 34 |
| Yes | 23 | 21 | 33 | 21 | -- | -- | -- | -- | 25 | 24 | 35 | 22 |

COPD= chronic obstructive pulmonary disease; GBS= Group B *Streptococcus*. If GBS was isolated from more than one clinical site, the most life-threatening or deepest site of infection was considered the ‘most-serious’ infected site. Rates are per 100,000 and were not calculated if the number of GBS cases was <10.

*Race was missing for 3 patients.

^†^Ethnicity was missing for 7 patients.

^‡^Underweight was defined as body mass index (BMI) <18.5, normal weight as BMI 18.5 to 24.9, overweight as BMI 25.0 to 29.9, and obesity as BMI ≥30.0; class 1 obesity was defined as BMI 30.0 to 34.9, class 2 obesity as BMI 35.0 to 39.9, and class 3 obesity as BMI ≥40.0; BMI was missing for 8 patients.
